# Clinical Lecture on Heart Disease in Children

**Published:** 1907-02-23

**Authors:** Carey Coombs

**Affiliations:** Physician to Out-patients, Children's Hospital, Bristol; Curator of the Museum, General Hospital, Bristol; Demonstrator of Pathology, University College, Bristol


### Hospital Clinics.

### CLINICAL LECTURE ON HEART DISEASE IN CHILDREN.

By CAREY COOMBS, M.D.(Lond.), Physician to Out-patients, Children's Hospital, Bristol; Curator of the Museum, General Hospital, Bristol; Demonstrator of Pathology, University College, Bristol.

My remarks will be based upon notes of two cases, widely differing in nature, yet each representing a type of cardiac disease prominent in, and almost exclusively belonging to, childhood.

The first case is that of G. L., 2½ years old, a gipsy child, sent as an out-patient to the Children's Hospital. The family history was unimportant. Since two months of age she had been dusky of complexion and very readily breathless. Recently the abdomen had been painful and, it was said, swollen; of this I found no evidence. After crying, or when she was lying down, the child was very cyanosed and breathless; in addition her fingers were "clubbed." Cardiac pulsation could be seen, and felt, extending from the anterior axillary line on the left, to the mammary line on the right, the maximum point being just to the left of the lower end of the sternum; the cardiac dullness was slightly outside this area in each direction. No thrill was felt. The sounds were dominated by a loud, long, rasping, systolic murmur heard with the greatest intensity over the upper and mid sternum and immediately to its right and left; it followed but did not replace the first sound, and the diastolic part of the cardiac cycle was free from bruit. Respiration and position had no influence upon the signs.

With symptoms beginning only two months from birth this child is clearly the victim of a congenital cardiac defect. Is it any good to try to state the nature of this defect? Sometimes such an attempt is possible and is helpful in prognosis. As about four-fifths of all cases clinically diagnosed as congenital heart disease are cases of pulmonic stenosis, a good way to proceed is to consider a case of congenital heart disease to be one of pulmonic stenosis unless the physical signs are very unlike those we usually find when the pulmonary artery is narrowed, or very like those definitely associated with other defects. In the few cases of pulmonic stenosis of which I have notes, the signs most constantly noted have been cyanosis, enlargement of the heart to the right as well as to the left, and a loud systolic murmur heard all over the præcordium (and often all over the chest), and loudest as a rule over the left upper chest. The only difference in the present case is the point at which the murmur is loudest, and this is not enough to set aside the diagnosis of pulmonic stenosis. Probably the pulmonic orifice is so narrow that blood also escapes from the right ventricle through a patency of the foramen ovale of the septum ventriculorum or of the ductus arteriosus.

The causation of pulmonary stenosis has been much debated; is it due to a purely developmental error, or is it a result of intra-uterine endocarditis? The truth is probably a compromise. Endocarditis in utero is not merely hypothetical (see Poynton's interesting examples, *British Medical Journal*, 1906, vol. i., p. 1458), but I should think it unlikely that the pulmonary valves are much more often the seat of the inflammation before birth than they are after. Without doubt, on the other hand, the arterial trunk itself may be narrow and even impervious by reason of incomplete development. The aorta and pulmonary artery are developed by division of the arterial bulb into two trunks; this is brought about by the formation of a longitudinal septum growing towards the heart. Sometimes the inter-arterial septum cheats the pulmonary artery, and sometimes (but very rarely) the aorta; the result is a pulmonary stenosis or even atresia. In the Bristol General Hospital Museum, for example, Specimen 252 shows a "pulmonary artery so small as barely to admit a crow-quill; the valve segments form a continuous ring with a central aperture." In specimen 255 the artery is quite impervious, and both ventricles empty into the aorta, the blood from the right ventricle doing so by passing through the space left by incomplete development of the basic portion of the interventricular septum. Further evidence of the developmental factor in congenital heart lesions is seen in the association of such lesions with other germinal defects; thus in a case referred to by me in the *St. Mary's Hospital Gazette* for July 1906, a child, the subject of mongolism and congenital cataract also showed signs of congenital pulmonic stenosis and of dilatation of the colon; and another child I have seen had an incomplete abdominal wall as well as pulmonary obstruction.

The diagnosis of such a case as the present one is not hard, but sometimes a child may be supposed to have a congenital cardiac defect when in reality there is no such thing. Cardio-pulmonary bruits at the basal areas, due to pulmonary fibrosis and collapse, may be misleading; but there are differential points, such as the influence of respiration and position on the signs. Again, over the heart of the very young infant a distinct systolic murmur may be heard, but this usually disappears by the second or third month.

What hope can be given to the parents of this child? Most of the subjects of pulmonic stenosis die before reaching the age of twenty, but not all. I have notes of a man of twenty-one, a porter, admitted to hospital for attacks of giddiness and fainting which had troubled him for a short time. He was dyspnœic and cyanosed, and there were unmistakable signs of pulmonic obstruction. In hospital he had several tetanic attacks, some of them apparently due to strychnine, and others not so easily explained. He improved on the whole and, I believe, went back to work. Another case was that of a woman, 36 years old, with symptoms dating from early childhood, and signs of pulmonary stenosis; she was admitted with œdema of the feet and attacks of præcordial pain. She was unable to undertake permanent work after leaving hospital. The very best that can be expected, then, is a life lasting till the third or fourth decade, with more than ordinary care; death is often due to some infection—tuberculosis, malignant endocarditis, and so on. In this particular instance there is such a degree of cyanosis that life is not likely to be long; cyanosis is evidence of deficient aeration of the blood, and its depth is therefore a good index of the degree of difficulty with which blood passes from the heart into the lesser circulation. Polycythæmia is often found (in this case it has not been looked for yet) and represents an attempt on the part of the organism to obviate the oxygen starvation of the tissues consequent on deficient aeration of the blood.

The second case I have chosen from a number of similar ones, because it is a good type of its class. N. H., a girl of nine, was brought to my out-patient department with chorea, the ninth attack; she began to suffer from it at the age of five, and at the same time had aching pains in the feet and ankles which have troubled her on and off since. At six she had an acute illness which, from the mother's description, may have been pericarditis, or pleurisy, or both. She has had many attacks of tonsillitis, and her tonsils are permanently enlarged (this, however, is by no means always the result of repeated inflammations). There was no family history of rheumatism, chorea, or sore throat. Apart from the chorea the only symptoms complained of when I first saw her were shortness of breath on exertion and tired attacks.

"The area of cardiac pulsation is very wide, reaching from the left anterior axillary line to a short distance beyond the right sternal margin in the third and fourth interspaces; the maximum point lies one inch outside the left mammary line in the fifth space. There is no thrill. The impulse is peristaltic in character, appearing first of all at the inner ends of the second and third left spaces, and passing obliquely downwards and outwards to the point of maximum intensity; at the time when the chest wall is being thrust forward at this latter point it is sinking in at the spots where the peristaltic wave started." This sinking in is not the same as the systolic recession described as occurring in cases of adherent pericardium. Another point appreciated by careful palpation is "a diastolic shock in the second left interspace synchronous with the pulmonary second sound. The cardiac dullness is increased transversely in both directions, reaching on either side a finger's breadth beyond the area of pulsation. At the apex the first sound is abnormally loud, ending in a long, blowing systolic murmur transmitted definitely into the axilla. The second sound is very distinctly doubled." Sometimes, in rather more advanced cases, the second half of the second sound is followed by a short, late diastolic murmur; this is not found in the child under consideration. "At the basic areas the systolic bruit is faintly heard, but the most striking alteration is a very great exaggeration of the pulmonary second sound."

Now this account of the physical signs of cardiac disease might apply not only to N. H. but to many other children the subjects of rheumatic infection. The transverse increase in both directions in the area of impulse and of dullness, proves dilatation of both ventricles; that the mitral valve is incompetent is shown by the apical bruit, and the consequence, hypertension in the pulmonary circuit, is attested by the sharp and violent closure of the pulmonic valve.

The peristaltic nature of the ventricular impulse is accounted for by the fact that the heart when dilated is brought into abnormally wide and intimate contact with the chest-wall, so that the normal peristaltic nature of systole is more easily appreciated by the clinical observer. So far, then, we have no sign of anything more than dilatation of both ventricles, sufficient to cause mitral leakage and pulmonary hypertension. Rheumatic infection reaches the heart by way of the coronary arteries In the pericardium the inflammatory process is characteristically rheumatic; it is marked by periodical recurrences, each of which leaves its permanent result in the form of adhesions. If these are not only intrapericardial, but also mediastino-pericardial, certain characteristic signs are produced, which are not present in this case, and the work of the heart is very seriously hampered. These grave cases of adhesive pericarditis are in my experience uncommon, at any rate as far as the rheumatic heart-disease of childhood is concerned. Adhesions which merely obliterate the pericardial sac are of secondary importance. In the myocardium, both parenchyma and stroma are influenced by the infective agent of rheumatism; in the former are found fatty deposit and other dystrophic changes, while the connective tissue shows areas of fibroblastic proliferation and leucocytic infiltration surrounding capillaries. There are signs of infection in the endocardium; within the mitral and aortic valves characteristic nodular swellings are formed, and the endothelium covering these is shed, fibrin being deposited in small quantity on the denuded surface. Very rarely, the inflammation may lead to rapid and unchecked destruction of tissue; the organisms (whatever they may be, probably the micrococcus found independently by various observers) thus find a ready access from the depths of the valve to the blood passing through the heart and scatter themselves and their poisons broadcast through the body. But more often the process is not ulcerative; the nodule disappears, leaving a scar, which is added to by subsequent attacks of inflammation, until, years later, the valves are found converted into hard unyielding plates of fibrous tissue.

In the heart of the child under discussion, where has the principal damage been done by the infection? Clearly not in the pericardium; there is no evidence of pericardial adhesion thick enough to account for the extreme degree of dilatation present. Neither is it to be found in the valves; for in childhood we meet with the beginnings of valvular disease, and it is not till years after the first infection of the cusps that their functions are seriously deranged. No; it is as we might on *a priori* grounds expect. That part of the heart which is of the greatest importance in health is the myocardium, and therefore an infection attacking all parts of the heart will produce its gravest effects when it interferes with the heart muscle. In the rheumatic carditis of childhood the muscular lesions are of the first importance, and indeed a fatal issue may prove post-mortem to have been due solely and entirely to myocarditis. I have recently made an autopsy upon a child who a few weeks before death was attending the out-patient department with signs closely similar to those we have already found in N.H.; he died, soon after admission, with tricuspid incompetence, probably due to a fresh instalment of myocardial rheumatism which proved "the last straw." Neither pericardium nor endocardium was diseased, but the heart was enlarged and globular in form, and the muscle taken from the apex of the left ventricle showed the evidences of interstitial inflammation. Rheumatic infection of the heart may also kill during childhood by producing (*a*) ulcerative endocarditis, (*b*) pericardial effusion, or (*c*) pericardial fibrosis; in the first case death is due to general infection, and in the other two to cardiac failure. Since myocarditis is also fatal in the same way as (*b*) and (*c*), in any given case of cardiac breakdown in a rheumatic child, the question to decide is whether myocarditis or pericarditis, with its consequences, is the principal factor, and it is safe to say that unless there is very distinct evidence of pericardial inflammatory exudation, of either sort, the heart muscle should be thought of as the more gravely affected tissue. Fortunately, fatal cases are not very common.

As for treatment, there is no space to deal fully with this; yet it may be remarked that the conception of rheumatic infection of the heart being of principal importance in its effects upon the myocardium, should make us insist more strongly than ever upon the tremendous importance of prolonged rest for children thus afflicted.

